# Development of a Sensitive *Escherichia coli* Bioreporter Without Antibiotic Markers for Detecting Bioavailable Copper in Water Environments

**DOI:** 10.3389/fmicb.2019.03031

**Published:** 2020-01-24

**Authors:** Yilin Pang, Xiaojun Ren, Jianghui Li, Feng Liang, Xiaoyu Rao, Yang Gao, Wenhe Wu, Dong Li, Juanjuan Wang, Jianguo Zhao, Xufen Hong, Fengying Jiang, Wu Wang, Huaibin Zhou, Jianxin Lyu, Guoqiang Tan

**Affiliations:** ^1^Zhejiang Provincial Key Laboratory of Medical Genetics, Key Laboratory of Laboratory Medicine, Ministry of Education, China, School of Laboratory Medicine and Life Sciences, Wenzhou Medical University, Wenzhou, China; ^2^College of Bioscience and Biotechnology, Hunan Agricultural University, Changsha, China; ^3^Laboratory of Molecular Medicine, Zhejiang Provincial Key Laboratory for Technology and Application of Model Organisms, Key Laboratory of Laboratory Medicine, Ministry of Education, China, School of Laboratory Medicine and Life Sciences, Wenzhou Medical University, Wenzhou, China; ^4^People’s Hospital of Hangzhou Medical College, Hangzhou, China

**Keywords:** whole-cell bioreporter, detecting bioavailable copper, gene knock-in, GFPmut2, *Escherichia coli*

## Abstract

The whole-cell bioreporters based on the *cop*-operon sensing elements have been proven specifically useful in the assessment of bioavailable copper ions in water environments. In this study, a series of experiments was conducted to further improve the sensitivity and robustness of bioreporters. First, an *Escherichia coli* △*copA*△*cueO*△*cusA* mutant with three copper transport genes knocked out was constructed. Then, the *copAp::gfpmut2* sensing element was inserted into the chromosome of *E. coli* △*copA*△*cueO*△*cusA* by gene knock-in method to obtain the bioreporter strain *E. coli* WMC-007. In optimized assay conditions, the linear detection range of Cu^2+^ was 0.025–5 mg/L (0.39–78.68 μM) after incubating *E. coli* WMC-007 in Luria–Bertani medium for 5 h. The limit of detection of Cu^2+^ was 0.0157 mg/L (0.25 μM). Moreover, fluorescence spectrometry and flow cytometry experiments showed more environmental robustness and lower background fluorescence signal than those of the sensor element based on plasmids. In addition, we found that the expression of GFPmut2 in *E. coli* WMC-007 was induced by free copper ions, rather than complex-bound copper, in a dose-dependent manner. Particularly, the addition of 40 mM 3-(*N*-Morpholino)propanesulfonic acid buffer to *E. coli* WMC-007 culture enabled accurate quantification of bioavailable copper content in aqueous solution samples within a pH range from 0.87 to 12.84. The copper recovery rate was about 95.88–113.40%. These results demonstrate potential applications of *E. coli* WMC-007 as a bioreporter to monitor copper contamination in acidic mine drainage, industrial wastewater, and drinking water. Since whole-cell bioreporters are relatively inexpensive and easy to operate, the combination of this method with other physicochemical techniques will in turn provide more specific information on the degree of toxicity in water environments.

## Introduction

Copper, one of the most common heavy metal elements, is an essential trace element for cellular metabolism. Research show that excessive copper for all living organisms is toxic (Karlsson et al., 2008). For instance, an excess of copper in the human body substantially contributes to the pathogenesis of Indian childhood cirrhosis (Freed et al., 2012), endemic Tyrolean infantile cirrhosis (Muller et al., 1996), and Wilson disease (Xu et al., 2019). In addition, accumulation of copper in the human brain can cause pathological changes in the nervous system, cerebellar motor dysfunction, and even Parkinson’s or Alzheimer’s disease (Emerit et al., 2004; White et al., 2006). It also has adverse effects on male fertility (Wirth and Mijal, 2010). In recent years, studies show that excessive copper may disrupt the labile [4Fe–4S] clusters in dehydrogenases (Macomber and Imlay, 2009) and block iron–sulfur cluster biogenesis in *Escherichia coli* (Fung et al., 2013; Tan et al., 2017) and *Bacillus subtilis* (Chillappagari et al., 2010).

Because the scale and pace of urbanization and industrialization have rapidly increased with the reform and opening-up policy of China, metal pollution has become a noticeable problem nowadays (Yang et al., 2018; Zhao Y. et al., 2018). In many cases, water environments close to human activity suffer from various degrees of copper pollution (Huang et al., 2013; Tang et al., 2013; Li F. et al., 2018), which threatens the water safety of local residents and causes huge economic losses. Therefore, prevention and control of copper pollution cannot be ignored, which is particularly important for detecting and monitoring toxic copper in the environment.

Currently, detection of heavy metal pollutants in the environment mainly relies on physicochemical methods, like atomic absorption spectroscopy (AAS) (Thongsaw et al., 2019) and inductively coupled plasma mass spectrometry (ICP-MS) (Fu et al., 2019). Although these methods are accurate, sensitive, and specific, they require expensive instrumentation and professional operation. Hence, it is difficult to apply at the grassroots level or in the field. Besides, quantification by these methods reflects the total copper content, including both bioavailable and non-bioavailable fractions (Martin-Betancor et al., 2015), making it difficult to objectively assess the toxicity of heavy metals. Therefore, developing an inexpensive, sensitive, selective, and portable method to determine the bioavailability of copper is necessary.

The academic definition of bioavailability is originally derived from the use of drugs, often associated with drug absorption in the human body or organs. It also applies to environmental pollutants (Impellitteri et al., 2003), but there is no clear definition so far (Li et al., 2014). In general, bioavailability of heavy metals is defined as those forms of metal that are absorbed and elicit an adverse effect on organisms (Deaver and Rodgers, 1996). As differences in metabolism and resistance mechanism of heavy metals exist among different species, bioavailability determined with one organism does not necessarily account for bioavailability for other organisms (Kang et al., 2018).

Whole-cell bioreporters have gained worldwide attention because they are sensitive, selective, portable, reasonably priced, and easy to handle. In addition, they have a relatively long life and a short response time (Fan et al., 2017; Jiang et al., 2017). Typically, a whole-cell bioreporter is consisted of a promoter–operator, which acts as the sensing element for heavy metal ions, with one or more reporter genes that encode easily detectable proteins (Jiang et al., 2017). However, to survive in high levels of heavy metal and reduce its toxic effect, bacteria have evolved a variety of heavy metal resistance mechanisms involved in the extrusion of metal ions from the cell (Leedjarv et al., 2008). These mechanisms lead to decrease in sensitivity of detection and unstable detection range of whole-cell bioreporters. Thereby, it is essential to elucidate the genetic mechanism of the response of host to specific metal to construct whole-cell bioreporters (Xu et al., 2013). In addition, the selectivity of typical whole-cell bioreporters is poor because of the non-specific cellular response to metal ions (Martin-Betancor et al., 2015; Din et al., 2019). For these reasons, the application of whole-cell bioreporters is restricted and in slow development.

**FIGURE 1:**
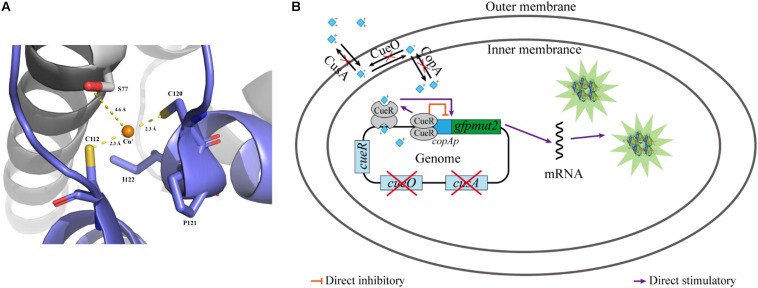
Diagram of working mechanism of the bioreporter *E. coli* WMC-007. **(A)** Cartoon representation of the binding site of Cu^+^ in CueR, and the copper-binding residues shown in stick representation. Polar interactions, stabilizing the position of Cu^+^ in the binding site, are indicated by yellow dashed lines. CueR is from *E. coli* K-12. This image was rendered using PyMOL (http://www.pymol.org); **(B)** schematic illustration of the bioreporter *E. coli* WMC-007.

*Escherichia coli* is one of the most common host strains used to construct whole-cell bioreporters for detecting copper ions (Riether et al., 2001; Hakkila et al., 2004; Ivask et al., 2009; Kang et al., 2018). *E. coli* has two copper efflux systems, the *cue* and *cus* system, to maintain a cellular demand level of copper: CopA, a P-type ATPase that pumps Cu(I) from the cytoplasm into periplasm (Rensing et al., 2000); CueO, an oxidase that oxidizes Cu(I) to Cu(II) in the periplasm to prevent adventitious Cu(I) entry into the cytoplasm again (Outten et al., 2001); and CusA, a subunit of a copper pump that transports copper ion from the periplasm to the extracellular environment (Munson et al., 2000). The *cue* system is controlled by the transcription factor CueR (Outten et al., 2000). As a member of the mercury resistance regulator MerR family, CueR contains two copper-binding cysteines (C112 and C120, as shown in Figure 1A) in its C-terminal metal-binding domain and forms homodimers. The activated CueR dimer binding two monovalent copper ions (holo-CueR) activates transcription of the *copA* and *cueO* genes by binding to their promoter regions, which induces torsional transformations in the DNA conformation. The apo-CueR (copper-free dimer) is also able to binding to the promotor region resulting in a tight DNA conformation, which represses *copA* and *cueO* expression (Figure 1B) (Outten et al., 2001; Philips et al., 2015; Bittner et al., 2017). Moreover, similar operons exist in *Pseudomonas putida* (Li et al., 2014) and *Pseudomonas fluorescens* (Kakinen et al., 2011).

Based on the above copper homeostatic systems, researchers have tried to construct various sensing elements, including *copAp::luxCDABE* (the linear detection range of Cu^2+^ is 3–30 μM) (Riether et al., 2001), pSLcueR/*copAp::luxCDABE* [limit of detection (LOD), 0.15 μM CuSO_4_] (Ivask et al., 2009), pSLcueR/*copAp::PpyWT* luciferase (LOD, 0.15 μM Cu^2+^) (Roda et al., 2011), and pPROBEcueR/*cueAp::gfp* (the detection range of Cu^2+^ is 1–70 mg/L) (Li et al., 2014), which were incorporated into the plasmids and transformed into the wild-type *E. coli* or *P. putida* to detect bioavailable copper. Recently, Kang et al. (2018) coexpressed the pCopAp-eGFP and pCDF-Duet-CueR plasmids in *E. coli* △*copA*△*cueR* mutant strain, significantly increasing its copper ion sensitivity. In particular, effectively reduced the background signal of whole-cell bioreporters by overexpressing CueR, thereby further enhancing sensitivity in response to copper ions (the detection range of Cu is 0–10 μM), compared with pCopAp-eGFP/*E. coli* △*copA*△*cueR* bioreporter. Our previous work reported a dual-functional *E. coli* cell that could detect and remove copper ions, which selectively responded to copper ions with a linear detection range of 0.01–25 μM (Wang et al., 2019).

The function of plasmid replication and segregation plays a key role in ensuring plasmid stability. Multiple factors can affect the stability of plasmid segregation, including the plasmid copy number, its replication mechanism, the levels of recombinant gene expression, the type of host strain, and the metabolic burden associated with all these factors (Sukhija et al., 2012; Kramer, 2016). Previous studies show that most recombinant plasmids are unstable and can be divided into two types. One is that the instability of segregation leads to the loss of plasmids; the other is the loss of heterologous genes caused by structural instability (Imanaka et al., 1980; Sukhija et al., 2012).

Even though a number of copper-induced whole-cell bioreporters with high sensitivity and/or strong specificity already exist, there are some drawbacks. In most cases, the reporter genes are harbored by the host strain in a medium or high-copy-number plasmid (Magrisso et al., 2008). Thus, in the process of bacterial cell division, it is potentially prone to copy number variability and loss of plasmids (Tom-Petersen et al., 2001). Moreover, high-copy-number plasmids could lead to unnatural and deleterious dosing of foreign genes, causing significant metabolic burden to host cells and leading to risks of horizontal gene transfer (Wrobel and Wegrzyn, 1998; Sukhija et al., 2012; Shemer et al., 2017). In particular, the background fluorescence value is very high without the addition of exogenous copper ions and overexpression of CueR (Kang et al., 2018). Nevertheless, overproduction of CueR and reporter proteins within cells potentially increases the metabolic burden. Therefore, it is necessary to construct a more effective bioreporter to minimize the influence of adverse factors mentioned above for more reliable test results.

In this report, we constructed a bioreporter strain *E. coli* WMC-007 with two genetic modifications. One of them was an *E. coli* △*copA*△*cueO*△*cusA* mutant strain with three Cu resistance-conferring genes knocked out, giving rise to its high sensitivity to copper ions. The other one was a *copAp::gfpmut2* transcriptional fusion integrated in the chromosome of *E. coli* △*copA*△*cueO*△*cusA* (Figure 1B). The optimal growth conditions of *E. coli* WMC-007 and its selectivity and the optimum detection linear range parameters were determined. Preliminarily, *E. coli* WMC-007 was applied to detect copper ions in water environment samples. The results suggest that the constructed *E. coli* WMC-007 may have the potential to supplement other physicochemical techniques for detecting bioavailable copper ions in acidic mine drainage, industrial wastewater, and drinking water.

## Materials and Methods

### Chemicals

DNA polymerase, restriction endonucleases, T4 DNA ligase, and all primers were purchased from/synthesized by Takara corporation (Dalian, China). Sucrose, ZnSO_4_⋅7H_2_O, MnSO_4_⋅H_2_O, and AgNO_3_ were obtained from Xilong Chemical Industry Corporation (Shanghai, China). Ni_3_SO_4_⋅6H_2_O, MgCl_2_⋅6H_2_O, and CuSO_4_⋅5H_2_O were purchased from Xinbao Fine Chemical Factory (Shanghai, China). HgCl_2_ was purchased from Beisite Chemical Reagent Corporation (Chengdu, China). 3-(*N*-Morpholino)propanesulfonic acid (MOPS), Pb(NO_3_)_2_, FeC_6_H_5_O_7_, (NH_4_)_2_Fe(SO_4_)_2_, and CoCl_2_⋅6H_2_O were purchased from Sigma-Aldrich (Shanghai) Trading Corporation. The standard copper solution (1,000 μg/ml) was obtained from the National Standard Substances Center (Beijing, China). Other chemicals used were of analytical grade. All the medium and buffer solutions were prepared using deionized distilled water (Millipore, United States). All tube cultures of bacteria were soaked in potassium dichromate solution for at least 24 h to remove residual heavy metals.

### Bacterial Strains, Plasmids, and Growth Conditions

Strains and plasmids used in this study are described in Supplementary Table S1. Genes encoding three major proteins (CpoA, CueO, and CusA) regulating intracellular copper homeostasis were deleted following the protocol developed by Datsenko and Wanner (2000). The gene deletion was confirmed by PCR. All primers for the deletion and confirmation can be found in Supplementary Table S2.

All experiments, unless stated otherwise, were conducted with the following protocol. *E. coli* WMC-007 was grown in Luria–Bertani (LB) at 37°C and rotated at 250 rpm. Overnight cultures were diluted to OD_600_≈0.02 using fresh LB medium and simultaneously adding the standard copper ion solution. The optical density at 600 nm (OD_600_) was determined with a Beckman Coulter DU800 spectrophotometer.

### Construction of Sensing Element and *E. coli* Bioreporters

The fused reporter gene, *copAp*::*gfpmut*2-*soxSCo*, was constructed with crossover PCR using three pairs of primers (Supplementary Table S2). The 3′ end of *PcopA*::*gfpmut*2 was fused with the transcription terminator of *soxS*. The PCR products were ligated into pET28a vector. The ligation mixture was transformed into DH5α. The bioreporter strains were constructed by transforming *copAp*::*gfpmut*2-pET28a into wild-type MC4100, *E. coli* △*copA*△*cueO*△*cusA*, and other single- and double-mutant strains.

The *gfpmut2* reporter gene knock-in was achieved following the procedure developed by Church’s group (Link et al., 1997). Briefly, the fused reporter gene, *copAp::gfpmut2*, was constructed with crossover PCR using three pairs of primers (Supplementary Table S2). The PCR products were cloned into a low-copy number and temperature-sensitive vector pKOV with chloramphenicol-resistant and sucrose-sensitive markers (Link et al., 1997). The cloned plasmid was introduced into *E. coli* △*copA*△*cueO*△*cusA* cells. The transformants were selected on LB plates containing 20 μg/ml chloramphenicol at 43°C. The obtained colonies were immediately plated on LB plates containing 5% (*w*/*v*) sucrose at 30°C to select the colonies that underwent the second recombination event. The colonies which were sucrose resistant and chloramphenicol sensitive were selected for further analysis. The positive colonies were confirmed with PCR using the primers flanking the target genes.

### Minimal Inhibitory Concentration Assay

First, overnight cultures were grown in fresh liquid M9 minimal medium at 37°C and rotated at 250 rpm for 2 h, then diluted to OD600≈0.005 using fresh M9 minimal medium before preparing 5-ml aliquots. Simultaneously, different concentrations of standard copper ion solutions were added to each aliquot to have a final concentration of 0, 0.125, 0.25, 0.5, 1, 2, 4, 8, 16, and 32 μM. The mixture was incubated at 37°C and 250 rpm for 2–10 h and monitored by cell density measurements at 600 nm every 2 h for three aliquots from each concentration.

### GFPmut2 Expression Kinetics Experiments

*E. coli* WMC-007 cells were grown in LB medium and incubated with 100 μM standard copper ion solutions at 37°C for 1–8 h. An equal volume of MilliQ H_2_O was added as blank control. All experiments, unless stated otherwise, were conducted with the following protocol. One milliliter of each sample was harvested, washed with pH 8.0 desalting buffer (contained 20 μg/ml chloramphenicol to block new GFPmut2 protein synthesis) twice, and diluted 20 times with the same buffer. Subsequently, the cell density of each sample was measured at 600 nm. The GFPmut2 assay was performed using a RF-5301 fluorescence spectrometer (Shimadzu Corporation, Japan) at an excitation wavelength of 481 nm and an emission wavelength of 507 nm.

### The Effect of Induction Time on the Sensibility and the Range of Linearity

*E. coli* WMC-007 culture was used to prepare 1-ml aliquot. Each aliquot was incubated with different concentrations of standard copper ion solutions at 37°C for 2–5 h. An equal volume of MilliQ H_2_O was added as blank control. Twelve aliquots were prepared for each concentration, and three of them were monitored every 1 h. The OD_600_ and GFPmut2 fluorescence intensity were measured as described above.

### Laser Confocal Microscopy Analysis

*E. coli* WMC-007 cells were harvested after incubation with different copper concentrations in LB medium for 5 h. The fluorescence of GFPmut2-producing cells was measured using a FluoView FV1000 laser confocal microscope (Olympus Corporation, Japan) with the following settings: excitation wavelength, 488 nm; voltage, 598 V; 1,024 × 1,024 pixel; magnification 600 times.

### Western Blot Analysis

The cell extracts were prepared by passing the cells through NS1001L2K high-pressure cell cracker twice. Cytosolic protein extracts (30 μg total protein per lane) were separated by sodium dodecyl sulfate polyacrylamide gel electrophoresis before blotting onto polyvinylidene fluoride membrane. GFPmut2 was detected using specific polyclonal antibodies from rabbit and goat antirabbit immunoglobulin G-conjugated with alkaline phosphatase as secondary antibody (Beyotime Institute of Biotechnology, China).

### Specificity and Selectivity Assay

The induction of the detection component by a variety of metal ions, including Ag^+^, Mg^2+^, Zn^2+^, Fe^2+^, Fe^3+^, Pb^2+^, Mn^2+^, Ca^2+^, Co^2+^, Ni^2+^, and Cu^2+^, was studied by measuring the green fluorescence produced. Each metal ion (5 μM) was added to *E. coli* WMC-007 cultures at a cell density of OD_600_ = 0.02. An equal volume of MilliQ H_2_O was added as blank control. The cells were incubated for 5 h at 37°C, and the specific fluorescence intensity was measured as described above. At least three independent experiments were performed for each kind of metal ion and mixtures of metal ion assays.

### Background Fluorescence Analysis

The experimental conditions of *E. coli* WMC-007 were as described above, except that MilliQ H_2_O was added instead of copper ion solution. For bioreporter *copAp::gfpmut2*-pET28a/*E. coli* △*copA*△*cueO*△*cusA*, namely, *E. coli* WMC-006, all experiments, unless stated otherwise, were conducted with the following protocol. The overnight cultures were diluted to OD_600_≈0.02 with LB medium, and cells were grown to OD_600_ = 0.5–0.6, which was used to prepare three aliquots of 1 ml. Ten microliters MilliQ H_2_O was added to each aliquot as blank control. Then, it was shake incubated at 37°C and 250 rpm for 3 h. Cells were washed with desalting buffer after centrifugation and resuspended to OD_600_ ≈ 0.25. The OD_600_ and the background fluorescence intensity was measured as described above.

### Flow Cytometry

For flow cytometric analysis of *gfpmut2* expression stability, the following procedure developed by Stiner and Halverson was carried out (Stiner and Halverson, 2002). The cultured cells were diluted with filtered (0.22-μm pore size filters) normal saline (NS) to approximately 5 × 10^6^ CFU/ml before analysis with a FACScan flow cytometer (BD, United States). The instrument was set to detect 10,000 cells for each sample.

### Effect of EDTA

The standard copper ion solution was added to each aliquot to reach a concentration of 1 mg/L. An equal volume of ethylenediaminetetraacetic acid (EDTA) in different concentrations was added simultaneously, giving final concentrations of 200, 400, and 800 μM. An equal volume of MilliQ H_2_O was added as blank control. The copper ion content in the sample was calculated according to the linear regression equation of the standard curve.

### Preparation of the Water Environment Samples for the Analysis With Bioreporter

Generally, water environments are highly acidic or alkaline when polluted by heavy metal ions. To minimize the influence of this environmental condition on bioreporter analyses, LB medium containing MOPS with a final concentration of 40 mM was used in this experiment (pH 7.2). One-tenth volume of aqueous solution with pH ranging from 0.87 to 12.57 was added to each culture aliquot. Simultaneously, standard copper solution was added to have a final concentration of 0.97 mg/L. The mixture was incubated for 5 h before analyzing the influence of pH on the copper recovery rate. The copper concentration in the sample was calculated based on the linear regression equation of the standard curve.

### Data Analysis

Unless otherwise stated, all data are presented as means ± SD.

Relative fluorescence unit (RFU) is defined as the ratio of the fluorescence intensity of bioreporter cells to the cell density at OD600; absolute fluorescence unit (AFU) is defined as the RFU of blank control subtracted from the RFU of the copper-exposed sample; induction coefficient (IC) is defined as the ratio of the relative fluorescence unit induced with copper ions to the background fluorescence unit of bioreporter cells without added heavy metal (Li et al., 2014; Kang et al., 2018).

The signal-to-noise (S/N) ratio was calculated as follows: S/N = (*F*_M_−*F*_DB_/OD600_M_)/(*F*_W_−*F*_DB_/OD600_W_), where *F*_M_ is the fluorescence intensity of bioreporter cells induced with metals, *F*_W_ is the background fluorescence intensity of bioreporter cells induced with MilliQ H_2_O, and *F*_DB_ is the fluorescence intensity induced with desalting buffer.

The limit of determination (LOD_AFU_) of copper for the bioreporter strains was determined following the procedure developed by Ivask et al. (2004). The calculation was based on this equation: LOD_AFU_ = 2(*X*_W_ + 3SD)/*X*_W_, where *X*_W_ is the mean background luminescence value of the bioreporter strains (10 blanks included in each assay) and SD is the standard deviation. The LOD as mol/L by the regression equation of the standard curve.

Bioavailable Cu^2+^ in water environment samples was determined using the Cu-inducible bioreporter *E. coli* WMC-007. The total Cu content was analyzed by AAS. Bioavailability was then calculated as the ratio of the copper concentration detected in bioreporters to the total copper content in water.

## Results

### Construction of the Bioreporters

**FIGURE 2:**
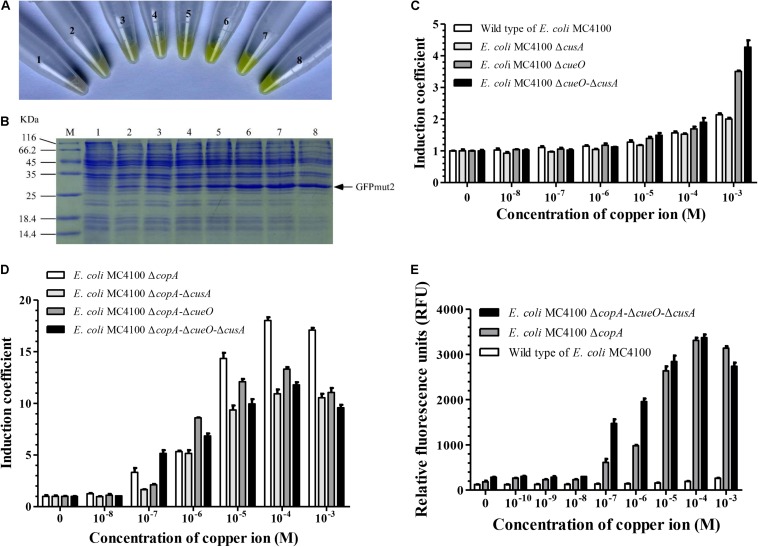
Effects of knockout of genes involved in copper transport in wild-type *E. coli* MC4100 on copper-sensing capability of bioreporters harboring *copAp::gfpmut2* sensing element. **(A)** The *copAp*::*gfpmut*2-pET28a/Δ*cusA-*Δ*copA*-Δ*cueO* (*E. coli* WMC-006) bioreporters induced by a series of copper concentration (numbers 2–8: 0, 1.0 × 10^–8^, 1.0 × 10^–8^, 1.0 × 10^–7^, 1.0 × 10^–6^, 1.0 × 10^–5^, 1.0 × 10^–4^, and 1.0 × 10^–3^ M copper), harvested and resuspended in Tris buffer. Number 1: pET28a/Δ*cusA-*Δ*copA*-Δ*cueO* as negative control. **(B)** Sodium dodecyl sulfate polyacrylamide gel electrophoresis (SDS-PAGE) (12%) analysis of whole-cell samples described in **(A)**. **(C,D)** Induction coefficient (IC) of copper-induced bioreporters with copper transport genes in the *cop*-operon knocked out. **(E)** Comparison of relative fluorescence intensity from wild-type *E. coli* MC4100, Δ*copA*, and Δ*copA*-Δ*cueO*-Δ*cusA* mutated strains harboring *copAp::gfpmut2* sensing element treated with different concentrations of copper ions. All the experiments producing Figure 2, were repeated at least once.

To improve the sensitivity of copper detection of *E. coli* MC4100, we constructed mutant strains of *E. coli* MC4100, in which one or several of copper response genes were knocked out. Minimal inhibitory concentration (Grass and Rensing, 2001) of the wild-type *E. coli* MC4100 and its mutants showed that *E. coli* △*copA*△*cueO*△*cusA* was the most sensitive to Cu^2+^. Its sensitivity was 64 times higher than that of the wild-type *E. coli* MC4100. The *copA* gene played a dominant role among the copper transport genes in *E. coli* (Table 1). Furthermore, when *E. coli* △*copA*△*cueO*△*cusA* cells were transformed with the *copAp*::*gfpmut*2-pET28a sensing element and induced by various concentrations of Cu^2+^, bright green color could be observed in the cell resuspension (Figure 2A). With increasing copper concentrations, the green color tended to be darker in a dose-dependent manner. To confirm that the accumulation of green color was due to the expression of GFPmut2, sodium dodecyl sulfate polyacrylamide gel electrophoresis analysis was carried out using the above whole cells and there were visible bands at ∼27 kDa in copper-induced samples (10^–3^–10^–8^ M), except for the blank control (Figure 2B). The amount of protein expression increased with copper concentration, which was consistent with the naked eye observation. These results indicated that the bioreporters were successfully constructed with high sensitivity.

**TABLE 1:** Minimal inhibitory concentration (MIC) of the wild-type *E. coli* MC4100 and its mutants.

Strain	MC4100	△*copA*	△*cueO*	△*cusA*	△*copA*−△*cueO*	△*copA* △*cusA*	△*cueO*−△*cusA*	△*copA*−△*cueO*−△*cusA*
MIC (μM)	16	0.25	8	16	0.5	0.25	4	0.25

The effects of the *E. coli* △*copA*△*cueO*△*cusA* mutant host cell on the response of *copAp*::*gfpmut*2 sensing element to copper was compared with *copA* single-mutant strain. We constructed eight whole-cell bioreporters with different mutant strains and tested their sensitivity to copper ions. As shown in Figures 2C–E, 1 mM copper ions induced weak fluorescence in bioreporter based on wild type of *E. coli* MC4100, as previously reported (Kang et al., 2018; Wang et al., 2019). The IC values suggested that the effect of copper transport genes on the sensitivity of these whole-cell bioreporters was ranked: △*copA* > △*cueO* > △*cusA* (Figures 2C,D). In the copper concentration range of 10^–7^–10^–6^ M, the IC of bioreporters based on *E. coli* △*copA*△*cueO*△*cusA* was significantly higher than that of *E. coli* △*copA*. However, when the concentration of copper was above 1 μM, the IC of bioreporters based on *E. coli* △*copA* was significantly higher than *E. coli* △*copA*△*cueO*△*cusA* (Figures 2C,D). To explain this contradiction, the responses of bioreporters based on *E. coli* △*copA* and *E. coli* △*copA*△*cueO*△*cusA* to copper within 0–1 mM were compared. The result indicated that the relative fluorescence signal derived from *E. coli* △*copA*△*cueO*△*cusA* was significantly stronger than that of *E. coli* △*copA*, while its background fluorescence intensity was 1.56 times higher than that of bioreporter *copAp::gfpmut2*-pET28a/*E. coli* △*copA* (Figure 2E). Similarly, our previous work showed that the fluorescent signal generated by the copper-sensing element in *E. coli* △*copA*△*cueO* was significantly stronger than that of *E. coli* △*copA* (Wang et al., 2019). Therefore, the mutant strain *E. coli* △*copA*△*cueO*△*cusA* was selected as the host strain to construct a new bioreporter by inserting the *copAp::gfpmut2* sensing element into its chromosome. This bioreporter was named *E. coli* WMC-007 and had the potential to reduce background fluorescence signal and accelerate the reaction rate.

### Time-Dependent Induction of the GFPmut2 Expression of the Bioreporter With Copper

**FIGURE 3:**
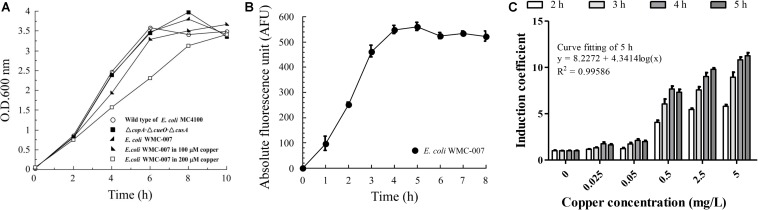
Optimization of incubation time for trace copper detection with bioreporter *E. coli* WMC-007. **(A)** Growth curves of the wild-type *E. coli* MC4100 (open circle), the *E. coli* Δ*copA*-Δ*cueO*-Δ*cusA* (closed squares), and the bioreporter *E. coli* WMC-007 incubated with (closed right oblique triangles and open squares) or without (closed left oblique triangles) different concentrations of copper in LB medium at 37°C for 0–10 h. **(B)** Induction kinetics of GFPmut2 treated with 100 μM (6.35 mg/L) copper. **(C)** Effect of induction time on the sensibility and the linear range of the bioreporter *E. coli* WMC-007. The data were obtained from three independent experiments.

The upper LOD is a key parameter limiting the application of whole-cell bioreporters. Before optimizing the kinetics of copper-induced GFPmut2 expression, it is necessary to determine the resistance limit of *E. coli* WMC-007. As shown in Figure 3A, the growth curves of the *E. coli* △*copA*△*cueO*△*cusA* and the *E. coli* WMC-007 were basically consistent with those of the wild-type *E. coli* MC4100. Cu^2+^ (100 μM; 6.35 mg/L) inhibited the growth of the *E. coli* WMC-007 slightly, while 200 μM Cu^2+^ significantly inhibited the growth of the *E. coli* WMC-007.

As shown in Figure 3B, under the condition of a fixed Cu^2+^ concentration, the trend of fluorescence accumulation in *E. coli* WMC-007 was almost consistent with its growth curve. After incubation for 4 h, GFPmut2 fluorescence intensity and the growth of *E. coli* WMC-007 entered the stationary phase. Further analysis showed that *E. coli* WMC-007 was able to produce detectable fluorescence signal after being exposed to copper ions for 2 h (Figure 3C). Moreover, the linear range and the IC of *E. coli* WMC-007 were significantly enlarged with the extension of incubation time. When induced for 5 h, the curve fitting coefficient *R*^2^ was closest to 0.9999, corresponding to the maximum detection sensitivity (Figure 3C). Therefore, 5 h incubation was selected as the best copper ion induction time, and the detection range of *E. coli* WMC-007 under this condition was 0.025–5 mg/L.

### Selectivity of Bioreporter *E. coli* WMC-007 to Heavy Metals

**FIGURE 4:**
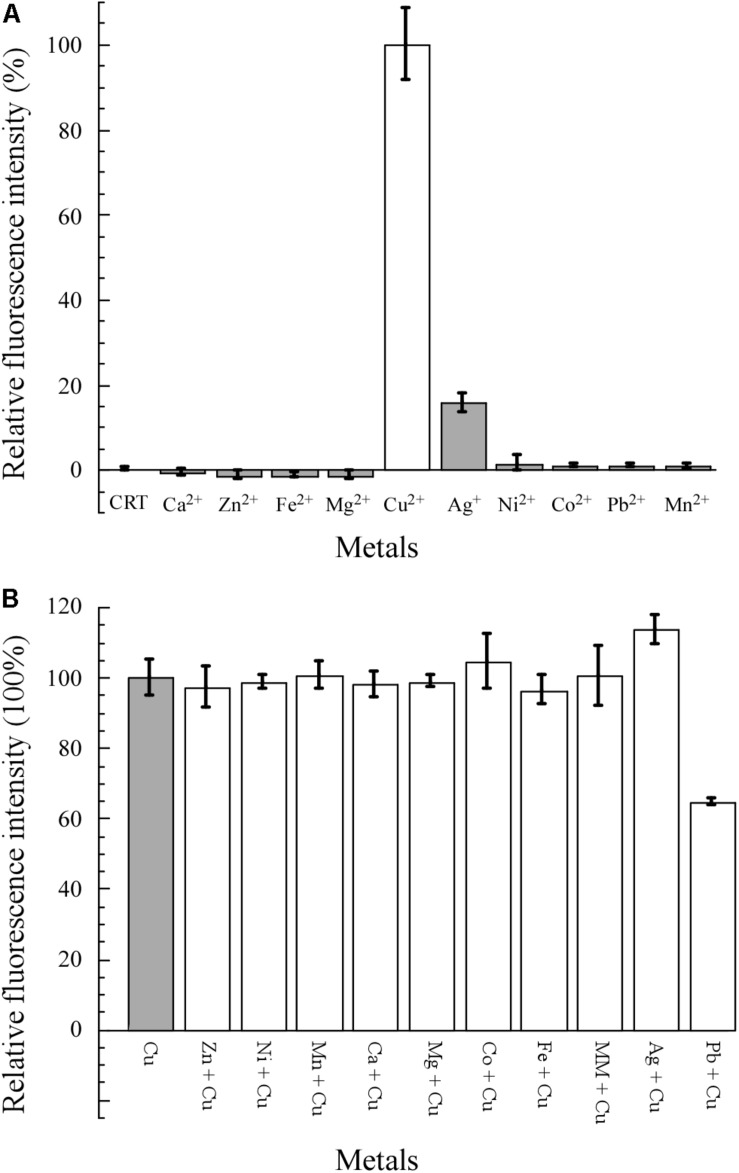
Specificity and selectivity of *E. coli* WMC-007 in response to different metals. MM, mixed metal, including all the metal ions shown in the figure except for lead and silver. The specificity **(A)** and selectivity **(B)** of *E. coli* WMC-007 for different metal ions (5 μM) were expressed in absolute fluorescence unit (AFU) ratio. The AFU value of Cu^2+^ was defined as 100% for reference. Data represent mean ± SD of three independent measurements.

As shown in Figure 4A, 10 common metal salts were used to test the specificity of *E. coli* WMC-007. Cu^2+^ was the only metal resulting in a statistically significant increase in the fluorescence signal. Ag^+^ could induce weak expression of GFPmut2 (the AFU value of Ag^+^ ≤ 20% of the AFU value of Cu^2+^) between 0.1 and 5 μM (data not shown), while other heavy metals demonstrated no obvious induction. The specificity of *E. coli* WMC-007 was consistent with those reported previously (Ivask et al., 2009; Kang et al., 2018; Wang et al., 2019).

The selectivity of *E. coli* WMC-007 for different metal ions was evaluated by performing pairwise metal assays. The final concentration of metal ions was 5 μM. As shown in Figure 4B, no statistically significant change in green fluorescence intensity was observed when mixing Mg^2+^, Zn^2+^, Fe^3+^, Mn^2+^, Ca^2+^, Co^2+^, or Ni^2+^ with Cu^2+^ separately, comparing with the control group (Cu^2+^). Same was observed when mixing all the metal ions. Interestingly, cross-reactions were detected when mixing Pb^2+^ and Cu^2+^, which gave rise to a significant reduction in the fluorescence intensity. By contrast, the green fluorescence was strengthened when mixing Ag^+^ and Cu^2+^. However, there was no such obvious cross-reaction in our previously reported bioreporter *E. coli* pBV-LOCG/Δ*copA*/*cueO* (Wang et al., 2019). It is speculated that the fluorescence signal generated by *E. coli* pBV-LOCG/Δ*copA*/*cueO* is 10 times as much as that of copper-induced *E. coli* WMC-007, so that the influence of Pb^2+^ and Ag^+^ on the fluorescence signal of pBV-LOCG/Δ*copA*/*cueO* can be ignored. Overall, the constructed *E. coli* WMC-007 has a high specificity and selectivity for Cu^2+^.

### Phenotype Characteristic of the *E. coli* Whole-Cell Bioreporters

**FIGURE 5:**
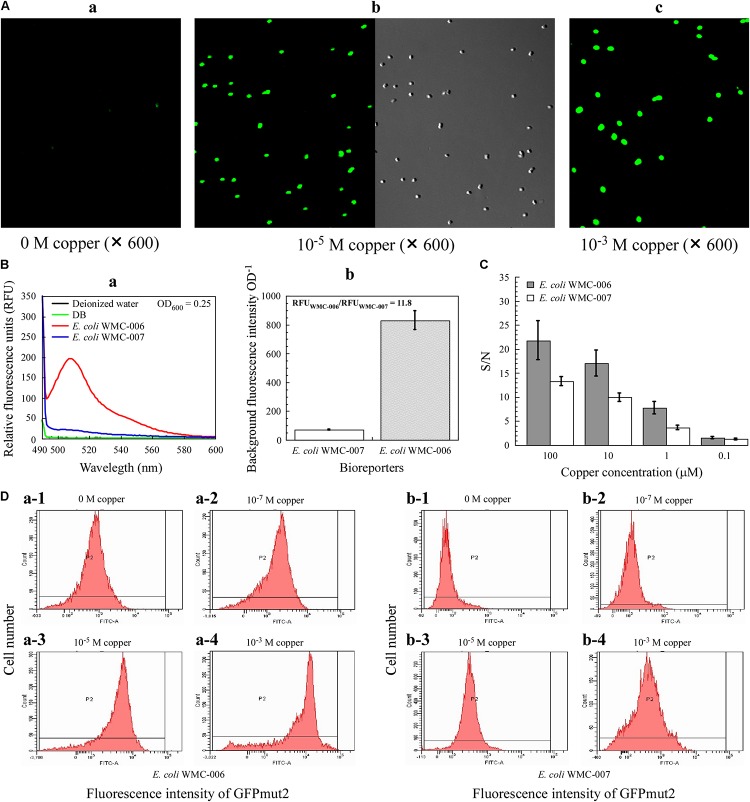
Phenotypic characteristics of *E. coli* bioreporters. **(A)** Laser confocal microscopy analysis of fluorescence intensity of the bioreporter *E. coli* WMC-007. **(B)** Comparison of background fluorescence produced by *E. coli* WMC-007 and *E. coli* WMC-006. **(C)** The signal-to-noise ratio (S/N) of bioreporters *E. coli* WMC-007 and *E. coli* WMC-006 in response to various copper concentrations. **(D)** Flow cytometry analysis of fluorescence signal homogeneity of the bioreporters. **(a,b)** Different types of peaks showing the distribution of log-transformed fluorescence intensity per cell upon exposure to different copper concentrations. The *y*-axis represents the number of fluorescent cells detected. The results presented in **(A–D)** were obtained from two and three independent experiments, respectively.

As shown in Figure 5A, under the excitation wavelength of 481 nm, the *E. coli* WMC-007 treated with 10 μM Cu^2+^ could emit bright green fluorescence, which was visualized through a confocal laser scanning microscope with blue light, further indicating a successful bioreporter construction. Fluorescence intensity of the cells also had dose-dependent effect with copper concentration, and the location of the *E. coli* WMC-007 cells emitting green fluorescence are consistent with the microscope’s FOV under natural light. Moreover, the fluorescence intensity of the *E. coli* WMC-007 was very uniform under the same copper ion concentration. These results demonstrated the reporter gene harbored by the host strain in the form of chromosomal integration enhancing the genetic stability of sensor element. On the other hand, when without addition of exogenous copper ions, the RFU produced by *E. coli* WMC-006 cells is about 12 times of the *E. coli* WMC-007, and the SD of three parallel measurements (*n* = 3) is also significantly larger than the *E. coli* WMC-007 (Figure 5B).

Furthermore, the S/N ratio for the detection of copper was compared between *E. coli* WMC-006 and *E. coli* WMC-007. As shown in Figure 5C, the SD value in *E. coli* WMC-007 experiment was significantly smaller than that in *E. coli* WMC-006 experiment. Nevertheless, the S/N value of *E. coli* WMC-007 was significantly lower than that of *E. coli* WMC-006 in the range of 1–100 μM copper. In addition, the difference in the S/N value became larger as the concentration of Cu^2+^ increased. Notably, when the concentration of Cu^2+^ dropped to 0.1 μM, there was no significant difference in the S/N value between the two bioreporters, while the SD in *E. coli* WMC-006 experiment was larger than that in the *E. coli* WMC-007 experiment.

### Analysis of the Robustness of Fluorescence Signal in Individual Bioreporter Cells

As shown in Figure 5D, the fluorescence intensity emitted by copper-exposed *E. coli* WMC-007 cells (5 h) exhibited a narrow unimodal distribution without long tails. By contrast, the fluorescence from *E. coli* WMC-006 cells followed a left-skewed unimodal distribution, particularly at 10^–3^ M copper. In addition, flow cytometry results showed that the fluorescence intensity of bioreporters increased with copper ion concentration. Moreover, the background fluorescence value of *E. coli* WMC-007 was significantly lower than that of *E. coli* WMC-006 (Figure 5D). These results demonstrated that the sensing element integrated into the chromosome is more robust than that integrated into plasmids.

### Dose-Dependent Induction of Green Fluorescence With Free Copper Ion, Rather Than Complex-Bound Copper

**FIGURE 6:**
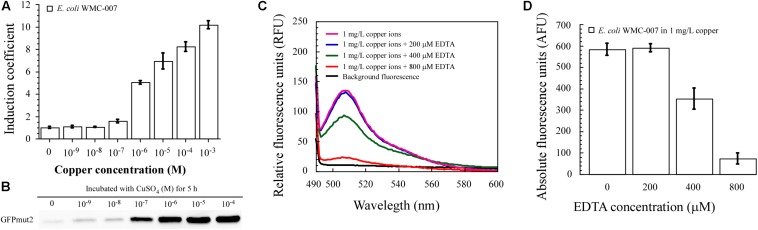
Dose-dependent induction of green fluorescence with free copper ion. **(A)** Induction coefficient (IC) of constructed bioreporter *E. coli* WMC-007 in response to 5-h incubation with Cu^2+^. **(B)** Western blot of GFPmut2 expressed by copper-induced *E. coli* WMC-007 after incubation for 5 h. **(C)** Fluorescence quenching effect of excess EDTA under the condition of a fixed copper concentration (1 mg/L). **(D)** Effect of excess EDTA on bioavailable Cu^2+^ content in aqueous solutions. At least two independent experiments were carried out for figure.

As shown in Figures 6A,B, the fluorescence intensity and expression level of GFPmut2 in *E. coli* WMC-007 cells were positively related to the free Cu^2+^ concentration. It is noted that only properly folded GFPmut2 can emit fluorescence. As a result, the linear relationship of GFPmut2 protein expression level after incubated with concentration gradient copper ions was not as good as the fluorescence intensity.

In conclusion, these results demonstrated that *E. coli* WMC-007 could accurately quantify the content of free copper ions in aqueous solution under laboratory conditions. However, copper can exist in many forms in water environment (Li A. et al., 2018). Our research findings suggest that the relationship between the total copper and the bioavailable copper in the aqueous solution can be dramatically changed by adding the chelator EDTA. As shown in Figures 6C,D, the fluorescence emitted by *E. coli* WMC-007 was not influenced by EDTA in low concentrations (0–200 μM). When the EDTA concentration was further increased, a large quantity of free Cu^2+^ were chelated in the medium. Consequently, the sensing element could not respond to EDTA-bound Cu since the chelated Cu could neither enter nor being available to *E. coli* cells. This experiment reveals that complex-bound copper affects the bioavailability of copper in water environment.

### Sensitivity of *E. coli* Bioreporters

**FIGURE 7:**
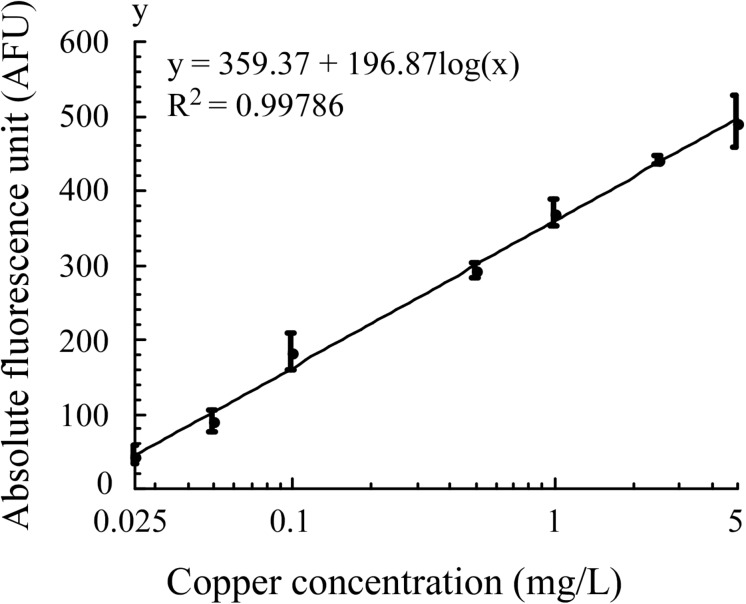
Fitted curve of absolute fluorescence unit produced by *E. coli* WMC-007 in response to copper in Luria–Bertani (LB) medium. Curve fitting was performed using KaleidaGraph software, with data obtained from at least three independent experiments.

In optimized assay conditions, the fluorescence intensity of *E. coli* WMC-007 exhibited a linear relation with the log10-transformed copper concentration at 0.025–5 mg/L (0.39–78.68 μM, *R*^2^ = 0.99786), and the LOD of Cu^2+^ was 0.0157 mg/L (0.245 μM), as shown in Figure 7. In comparison, the linear range in *E. coli* WMC-006 experiment was 0.005–2 mg/L (0.078–31.53 μM), and the LOD of Cu^2+^ was 0.0049 mg/L (0.0766 μM) (data not shown). These results show that the upper limit for copper detection with *E. coli* WMC-007 is about 2.5 times higher than that with *E. coli* WMC-006, while its lower limit is 5 times higher than that with *E. coli* WMC-006.

### Analytical Applications

To demonstrate the utility of *E. coli* WMC-007, actual water environment samples with a known concentration of contaminant were examined. Heavy metals are commonly found in extremely acidic environments, like mine drainages and acidizing tailings (pH < 3). To reduce the influence of extreme environmental conditions on the performance of *E. coli* WMC-007, pH buffer MOPS was added to LB medium at a final concentration of 40 mM (Riether et al., 2001; Hansen et al., 2019). Comparing with the copper concentration in aqueous solution samples within a pH range from 0.87 to 12.84 measured by AAS method, the copper recovery rate measured by *E. coli* WMC-007 turned out to be 95.88–113.40%, as listed in Table 2. Sample 2 (pH = −0.30) and sample 6 (pH = 14.70) had the highest and the lowest copper recovery, respectively.

**TABLE 2:** The recovery test results of copper ion isolated from acid-base samples.

Sample	pH^1,a^	pH^2, b^	Cu^2+^ added^d^ (mg/L)	Cu^2+^ found^e^ (mg/L)	Recovery (%)
1^c^	7.12	7.12	0.97	1.07 ± 0.0250	110.31
2	–0.30	6.73	0.97	1.18 ± 0.1283	121.65
2	0.87	7.08	0.97	1.06 ± 0.0189	109.28
4	10.80	7.11	0.97	1.10 ± 0.0234	113.40
5	12.84	7.13	0.97	0.93 ± 0.2159	95.88
6	14.70	7.52	0.97	0.68 ± 0.0035	70.10

*^*a*^Where pH^1^ is the pH of the acid-base samples. ^*b*^pH^2^ is the pH of the LB medium mixed with acid-base samples and 40 mM MOPS. ^*c*^Sample 1 is the blank control. ^*d*^The analysis result of the Cu^2+^ added column were measured by the AAS method. ^*e*^The analysis result of the Cu^2+^ found column were measured by the *E. coli* WMC-007.*

## Discussion

In this study, to reduce the detection threshold, three major copper efflux transporters were knocked out from the wild-type *E. coli* MC4100, so that trace copper ions could accumulate in the cell. The *copAp::gfpmut2* sensing element was inserted into the chromosome of *E. coli* △*copA*△*cueO*△*cusA* by knock-in method, producing a new bioreporter superior in a few aspects. To begin with, the gene knockout technique increased the sensitivity of wild-type *E. coli* MC4100 to copper ions by 64 times (Table 1), which improved the copper detection accuracy at low concentrations (nmol–μmol); second, there is no antibiotic marker gene in the single-copy sensor element, which reduces the background fluorescence signal under non-induced conditions (Figures 5A,B,D); Finally, the sensor element can be stably inherited with bacterial reproduction, which enhanced the robustness and reproducibility of the detection of copper ions (Figures 5A,C,D). Our results collectively demonstrated that the bioreporter *E. coli* WMC-007 has potential applications in determining Cu^2+^ in drinking water, irrigation water, and industrial wastewater according to the national standards of water quality in China (Figure 7 and Table 3).

**TABLE 3:** All kinds of national standards for water quality limits for copper in China.

Standard name	Limits (mg/L)				
Drinking water sanitary standard (GB5749-2006)	1.0				

	**I class**	**II class**	**III class**	**IV class**	**V class**

Quality standard for surface water (GB3838-2002)	0.01	1.0	1.0	1.0	1.0

	**I class**	**II class**	**III class**	**IV class**	**V class**

Quality standard for ground water (GB/T14848-93)	0.01	0.05	1.0	1.5	>1.5
Water quality standard for fishery (GB11607-89)	0.01				

	**I class**	**II class**	**III class**	**IV class**	

Sea water quality standard (GB3097-1997)	0.005	0.01	0.05	0.05	
Water quality standard of irrigation (GB5084-2005)	1.0				

	**I class**	**II class**	**III class**		

Integrated pollutant discharge standard (GB8978-1996)	0.4	1.0	2.0		

Green fluorescent protein (GFP) is a generally fluorescent protein and has a variety of advantages for applications. It is non-toxic, stable, and convenient for detection. Furthermore, its fusion expression is easy to achieve and can be monitored using flow cytometry and fluorescence microscopy (Stiner and Halverson, 2002; van der Meer et al., 2004; Chalfie, 2009; Roda, 2010). Therefore, whole-cell bioreporters based on GFP have been applied to detect a number of environmental toxicants, such as genotoxic compounds (Kostrzynska et al., 2002), toluene and related compounds (Li et al., 2008), endotoxin (Goh et al., 2002), and heavy metals (Liao et al., 2006; Cerminati et al., 2015). However, the wild-type GFP has weak fluorescence. If *lux* or *luc* reporter genes are replaced by *gfp* reporter gene, the sensitivity of bioreporters will be reduced even in the same conditions (Hakkila et al., 2002; Stocker et al., 2003).

In this study, we used *gfpmut2* (GFPmut2, S65A, V68L, S72A) reporter gene for detecting copper ions because the fluorescence signal produced by GFPmut2 was 100 times higher than that of the wild-type GFP. At high expression levels, GFPmut2 produces bright green fluorescence when stimulated by blue light (481 nm) alone. Therefore, it is possible to lower of the detection threshold of bioreporters, compared with GFP. This is confirmed by our study, demonstrating that *E. coli* WMC-006 and *E. coli* WMC-007 cells displayed yellow-green color after incubation with trace copper for 1–5 h (Figure 2A).

However, there are some drawbacks, including relatively weak fluorescence intensity, large S/N ratio, and higher LOD of copper. This is attributed to the single-copy sensor element in the chromosome. With the same incubation time, the amount of GFP produced is significantly less than that in *E. coli* WMC-006. A previous study showed that it took about 1–2 h for the cells to complete posttranslational fluorophore formation once GFP was formed (Stiner and Halverson, 2002). Therefore, we simultaneously added the standard copper ion solutions to the medium while inoculating. This method is not only convenient and time saving but also facilitates the accumulation of fluorescence signals. Moreover, the induction of GFPmut2 expression with copper ions was time and dose dependent, which indicated that the IC of bioreporters was positively correlated with the expression level of GFPmut2, in addition to the background fluorescence intensity (Figure 3C).

In conclusion, we speculate that increasing the copy number of the sensing element in the chromosome could effectively increase the S/N value of bioreporters and lower the detection threshold, under the condition that the background fluorescence signal of *E. coli* WMC-007 and the metabolic burden to host cells are not significantly increased, so that LOD of bioreporters can cover the copper detection limit in any circumstances according to Chinese national standards for water quality (as shown in Table 3).

Water environmental samples, especially wastewater effluents, are complex mixtures. Some components, such as EDTA and other “EDTA-like” substances, are known to bind metal ions, which may influence metal bioavailability in an unpredictable manner (Knepper et al., 2005; Peters et al., 2014; Constantino et al., 2017). Recent data from 162 wastewater treatment effluents in the United Kingdom reveal the median EDTA concentration of 128 mg/L (0.44 mM), which is 5.3 and 22.9 times greater than those of dissolved zinc and copper, respectively (Constantino et al., 2017). We also confirmed that EDTA-bound copper affects the bioavailability of copper in aqueous solution (Figures 6C,D). Moreover, Riether et al. (2001) reported that the luminescence of bioreporter MG1655 (*copAp::lux*) was significantly inhibited, when the EDTA/Cu^2+^ molar ratio exceeded 1. However, when the Na_2_S/Cu^2+^ molar ratio was above 1.25, the IC of MG1655 (*copAp::lux*) increased significantly.

In addition, Zhao C. et al. (2018) conclude that dissolved organic matter in urban stormwater runoff has a strong binding affinity with Cu^2+^, which may further lead to potentially significant influence on migration, transformation, and bioavailability of copper. Hu et al. (2017) also show that the changing composition of impoundment-derived dissolved organic matter can decrease the bioavailability and toxicity of Cu in Hongze Lake in China. Effluent organic matter from industrial and sewage wastewaters can be separated into hydrophobic, transphilic, and hydrophilic fractions depending on its polarity. Yoo et al. (2016) suggest that the binding and toxicity of copper are largely dependent on the polarity of effluent organic matter. Käkinen et al. (2016) also proved the solubility-driven toxicity of CuO nanoparticles to Caco2 and *E. coli*.

It is worth noting that the organic matter in LB medium may form complex with copper ions. We speculate that copper is easily released from the complex under acidic conditions, so that the concentration of free copper ions increases. When the medium becomes alkaline, copper ions are easily precipitated and difficult for bacteria to uptake, thereby reducing its bioavailability (as show in Table 2). These results demonstrate the potential application of the constructed *E. coli* WMC-007 in determining the bioavailability of copper ions with high sensitivity and accuracy in water environment samples. Most importantly, these results suggest that the results quantified by physicochemical analysis methods reflect the total copper content, making it difficult to objectively evaluate the toxicity to bacteria and human.

Generally, the composition of environmental pollutants is very complex. Nutrients, toxicants, and particles in the sample matrix may stimulate or quench the fluorescence of the bioreporters (Ivask et al., 2004). Therefore, in a follow-up experiment, we will construct a constitutively fluorescence control strain (Liao et al., 2006; Ivask et al., 2009) to cancel out the effects of the above factors on bacterial growth and the expression of GFPmut2. Like *E. coli* WMC-007, this constitutively fluorescence control strain is derived from *E. coli* △*copA*△*cueO*△*cusA* host cell with the expression of GFPmut2 or other fluorescent proteins. However, it lacks the metal recognizing protein and its corresponding promoter (Ivask et al., 2004). Its reporter gene is controlled by constitutive promoters, and if the promoter is a strong promoter will be perfect. Such as *lac*_*p*_::*gfpmut2*/MC4100 △*lacI*△*lacZYA*△*copA*△*cueO*△*cusA* whole-cell bioreporter.

Despite the fact that many whole-cell bioreporters have been constructed for measuring heavy metals, they have been rarely applied to monitor water environmental samples (Hynninen et al., 2010). Another obstacle to apply this technology is that even in highly contaminated samples, the amount of bioavailable metal in complex matrices mostly ranges between 0.1 and 50% (Magrisso et al., 2008). In our previous work, the bioreporter *E. coli* pBV-LOCG/ΔcopA/cueO was applied to detect copper in 117 water environmental samples. The average accuracy of the whole cell for copper quantification was 93.8 ± 30.1% compared to ICP-MS, despite the fact that the samples were acidified to pH of 2 using nitric acid (Wang et al., 2019). Therefore, these results prove that the whole-cell bioreporter is a reliable tool for high-throughput screening of copper contamination in environmental samples, although further research is needed to verify whether the bioavailable copper content in environmental samples can be truly reflected. In addition, the metal-responding element CueR is not specific for Cu, as it can be induced by Ag in relatively high concentrations (Ivask et al., 2009), which is supported by our finding in this study (Figure 4).

To solve these problems, we make several suggestions to improve *E. coli* WMC-007. (1) Like the Cd-specific mutants of mercury-sensing regulatory protein MerR constructed by Hakkila et al. (2011), oligonucleotideite-directed mutagenesis (Hakkila et al., 2011; Shemer et al., 2017) can be applied on the key metal-binding regions of Cu/Ag-sensing transcriptional regulatory protein CueR (mutated CueR, CueR-m). Besides, a double-promoter model will be used in the construction of the sensing element *copAp::cueR-m-copAp::gfpmut2*. This strategy may eliminate the response of the sensing element to Ag and further decrease the background fluorescence signal, thereby improving the specificity and sensitivity of the bioreporter. (2) Because the LOD and S/N ratio of *E. coli* WMC-007 are not as good as the *E. coli* WMC-006, one to two copies of *copAp::cueR-m-copAp::gfpmut2* sensing elements can be inserted into the position of *cueO* and/or *cusA* in the chromosome of *E. coli* WMC-007 to further improve the detection thresholds. (3) As mentioned above, a cell viability internal control bioreporter that constitutively expresses GFPmut2 can be constructed (Roda et al., 2011). (4) An optimal medium should be selected for ecotoxicological and microbiological tests (Kakinen et al., 2011). For example, our previous work demonstrated that the limit of whole-cell copper detection in M9S medium [M9 minimal medium supplemented with 0.2% glucose, 5 μg/ml thiamin, and the 20 amino acids (10 μg/ml each)] was significantly lower than that in LB medium (Wang et al., 2019). (5) The bioassay may be performed in 96- or 384-well black polystyrene microtiter plates for high-throughput testing. (6) The whole-cell bioreporter with a physicochemical transducer (for example, a digital microfluidic diluter chip) and a detector element can be combined to construct microbial biosensors for the application of *in situ* and real-time detection or monitor of copper in water environments (Shemer et al., 2017). (7) To be recognized by third-party evaluation agencies, it is necessary to standardize the testing process and establish a classification system and grading standard for bioavailable copper in water environments with the combination of AAS or ICP-MS methods.

## Data Availability Statement

The datasets generated for this study are available on request to the corresponding author.

## Author Contributions

GT and JXL conceived and supervised the study. GT, JXL, YP, DL, and WWu designed the experiments. YP, XJR, FL, XYR, YG, JW, JZ, JHL, and XH performed the experiments. YP, XJR, and FJ analyzed and interpreted the data. YP, GT, JHL, WWa, and HZ wrote and revised the manuscript. GT reviewed the manuscript.

## Conflict of Interest

The authors declare that the research was conducted in the absence of any commercial or financial relationships that could be construed as a potential conflict of interest.
