# Supplementary material for: Development of a Sensitive Escherichia coli Bioreporter Without Antibiotic Markers for Detecting Bioavailable Copper in Water Environments
Source: Front Microbiol. 2020 Jan 24;10:3031. doi: 10.3389/fmicb.2019.03031 (PMC6993034; doi:10.3389/fmicb.2019.03031)
Supplement: Supplementary file 1 [file Data_Sheet_1.pdf]

Supplementary Material

Supplementary Tables

Supplementary Table S1 Bacterial strains and plasmids

Strain or plasmid	Characteristics	Source or reference
Escherichia.coli strains		
MC4100	F^-, *araD*139, Δ(*argF-lac*), U169, *rspL*150, *relA1*, *flbB*5301, *fruA*25, *deoC*1, *pstF*25	CGSC
DH5α	F^-, λ^-, *endA*1, *hsdR*17, *hsdM*$^+$, *supE44*, *thi*1, *recA*1, *gyrA*96, *relA*1, Δ(*argF*, *lacZYA*), U169, φ80d, Δ(*lacZ*), M15	Stratagene
WMC4100.1	MC4100 Δ*copA*	This study
WMC4100.2	MC4100 Δ*cueO*	This study
WMC4100.3	MC4100 Δ*cusA*	This study
WMC4100.12	MC4100 Δ*copA*-Δ*cueO*	This study
WMC4100.13	MC4100 Δ*copA*-Δ*cusA*	This study
WMC4100.23	MC4100 Δ*cueO*-Δ*cusA*	This study
WMC4100.123	MC4100 Δ*copA*-Δ*cueO*-Δ*cusA*	This study
WMC-006	MC4100 *copAp*::*gfpmut2*-pET28a/WMC4100.123	This study
WMC-007	MC4100 *copAp*::*gfpmut2*/ WMC4100.123	This study
Plasmids		
pKD46	Amp^r, repA101(ts), araBp-gam-bet-exo, oriR101, bla(ApR), araC bla [tL3]	CGSC
pCP20	Cm^r, yeast Flp recombinase gene, temperature sensitive replication	CGSC
pKOV	Cm^r, temperature sensitive pSC101 replication origin-*repA*^{ts}, *sacB*	Harvard
pMD19-T	Amp^r, TA cloning vector, pUC origin	Takara
pET28a	$Kana^r$, Bacterial expression vector, T7 lac promoter, adds N-terminal His tag	SGC
PcopA::*gfpmut2*-pET28a	$Kana^r$, GFPmut2 expression vector, *PcopA* promoter, *SoxS-Co* terminator	This study

Supplementary Table S2 The primers used to construct the whole-cell bioreporters

Primer	Sequence (5′→ 3′)[a,b]
CopA-H1P1	AGGTTTAACCTTTATCACAGCCAGTCAAAACTGTCTTAAAGGAGTGTTTT GTGTAGGCTGGAGCTGCTTC
CopA-H2P2	CGTCGATGCCAAATGCGCCACCCTAAAGCAGCGCATCCGCAATGATGTAC CATATGAATATCCTCCTTA
CueO-H1P1	ATGCTCAACGTTTGATTTTGTTTCGCCTGCTTAAGAATAAGGAAATAACT GTGTAGGCTGGAGCTGCTTC
CueO-H2P2	ATCAGTTTAATGCCCGGAGAGATCCGGGCATATTTCCGAATACGGTCTTT CATATGAATATCCTCCTTA
CusA-H1P1	GAGCGGATGCGCTCTGAAAGTGCTACCCATGCGCATTGAGGGAATAACCA GTGTAGGCTGGAGCTGCTTC
CusA-H2P2	GCCCATCGTCGCAAGACACAATCCACACGGTTAAACGGGGTATCCTGCTT CATATGAATATCCTCCTTA
CopA-No	AAGGAAAAAAGCGGCCGCATCTTCTAACGCAAGGGCTAACG
CopA-Co	CGCACGCATGTCGACTGAGATGGCATTTCGTCTGGC
CueO-No	AAGGAAAAAAGCGGCCGCGCAAGCAGGCTTAAGGAATCG
CueO-Co	CGCACGCATGTCGACCCCGATGCCGGTTCC
CusA-No	AGCTTTGTTTAAACCTGCTGCGATCCCGGAG
CusA-Co	CGCGGATCCATCCGGGAACCAGTACTGCATATA
PcopA-1	GGGAATTCCTCACCCCGGTGCCG
RPG-2	GTGAAAAGTTCTTCTCCTTTACTCATAAAACACTCCTTTAAGACAGTTTTG
RPG-3	CAAAACTGTCTTAAAGGAGTGTTTTATGAGTAAAGGAGAAGAACTTTTCAC
SoxS-Co	CGCACGCATGTCGACCGATTTTCATACGTCACGGTTGATAG
P_1 (copA-No)	AAGGAAAAAAGCGGCCGCATCTTCTAACGCAAGGGCTAACG
P_2 (copA-Ni)	GTGAAAAGTTCTTCTCCTTTACTCATAAAACACTCCTTTAAGACAGTTTTGAC
P_3 (gfpmut2-N)	GTCAAAACTGTCTTAAAGGAGTGTTTTATGAGTAAAGGAGAAGAACTTTTCAC
P_4 (gfpmut2-C)	GCGCATCCGCAATGATGTACTTATTTGTATAGTTCATCCATGCCATG
P_5 (copA-Ci)	CATGGCATGGATGAACTATACAAATAAGTACATCATTGCGGATGCGC
P_6 (copA-Co)	CGCACGCATGTCGACTGAGATGGCATTTCGTCTGGC

[a] Underlined sequence is homologous arm sequence
[b] Double underlined sequence is restriction sites
